# Comparison of four sarcopenia screening questionnaires in community-dwelling older adults from Poland using six sets of international diagnostic criteria of sarcopenia

**DOI:** 10.1371/journal.pone.0231847

**Published:** 2020-04-20

**Authors:** Roma Krzymińska-Siemaszko, Sławomir Tobis, Marta Lewandowicz, Katarzyna Wieczorowska-Tobis

**Affiliations:** 1 Department of Palliative Medicine, Poznan University of Medical Sciences, Poznan, Poland; 2 Department of Geriatric Medicine and Gerontology, Poznan University of Medical Sciences, Poznan, Poland; Hong Kong Polytechnic University, HONG KONG

## Abstract

**Introduction:**

There are four screening sarcopenia questionnaires (SARC-F, SARC-CalF, MSRA-5, MSRA-7). To unambiguously determine which of them is the most effective tool in community-dwelling older adults, we performed a diagnostic accuracy study. The aim of the analysis was to assess the diagnostic values of SARC-F, SARC-CalF, MSRA-5, MSRA-7 and compare their psychometric properties against six criterion standards (EWGSOP1, EWGSOP2, FNIH, AWGS, IWGS, SCWD criteria).

**Materials and methods:**

We included 100 community-dwelling volunteers aged ≥ 65yrs. The sensitivity/specificity analyses were performed. Receiver operating characteristic (ROC) curves and area under the ROC curves (AUC) were calculated to compare the overall diagnostic accuracy of the four questionnaires. Ideal screening tools should have reasonably high sensitivity and specificity, and an AUC value above 0.7.

**Results:**

With respect to the six criterion standards used, the sensitivity of SARC-F, SARC-CalF, MSRA-5, and MSRA-7 ranged 35.0–90.0%, 20.0–75.0%, 64.7–90.0%, 76.5–91.7%, respectively, whereas the specificity ranged 86.9–91.1%, 80.0–90.0%, 45.8–48.8%, 28.9–31.0% respectively. The AUCs of SARC-F, SARC-CalF, MSRA-5, and MSRA-7 ranged from 0.655–0.882, 0.711–0.874, 0.618–0.782 and 0.588–0.711 respectively. Only SARC-CalF had AUC >0.7 and <0.9 against the six criterion standards but obesity was a confounding factor, which may affect the diagnostic power of SARC-CalF. MSRA-7 had the smallest AUC of all the questionnaires and MSRA-5 had slightly larger AUC than MSRA-7.

**Conclusion:**

Based on our analysis, the standard sarcopenia screening questionnaires deliver contradictory results in many practically occurring cases. It appears that SARC-CalF is an optimal choice for screening sarcopenia in community-dwelling older adults.

## Introduction

Sarcopenia is a significant public health concern which causes a substantial economic burden. Mijarends et al. [1] found that the average costs of health care provided to Dutch community-dwelling older adults with sarcopenia were almost three times higher than in non-sarcopenic individuals, amounting to 4,325 euro and 1,533 euro, respectively. It is thus essential to detect sarcopenia at the earliest possible stage, when there are yet no apparent symptoms of the condition (e.g. muscle weakness), to limit these over-expenses. Timely recognition of sarcopenia makes early treatment possible which, in turn, minimises the risk of severe consequences in the future (e.g. falls, injuries, hospitalisation, and even death) [2].

Despite the widespread interest in sarcopenia for over three decades (the term ‘sarcopenia’ was first proposed in medicine in 1988 by Rosenberg [3]), there exists no effective screening tool for this condition. The SARC-F questionnaire developed by Malmström and Morley, and first published in 2013, appears to be the most popular screening test [4]. A range of studies has found SARC-F to be characterised by low sensitivity but high specificity [5–8]. It is stressed, though, that high sensitivity is hugely desirable for a screening test, resulting in a good ability to detect individuals who actually have the condition. Given the low sensitivity of SARC-F, Barbosa-Silva et al. [9] proposed a new questionnaire, called SARC-CalF, in 2016. It evaluates the same domains as SARC-F, but it uses calf circumference (CC) as an additional measurement. In a few studies, SARC-CalF was found to have superior sensitivity than SARC-F, and similar specificity [9–11]. Another promising questionnaire proposed for sarcopenia screening is Mini Sarcopenia Risk Assessment Questionnaire (MSRA), available in two versions: short (MSRA-5) with five items, and full (MSRA-7) with seven items. The questionnaire, developed by Rossi et al. [12], was first published in 2017. However, the number of studies on its diagnostic value is minimal [12,13].

As already mentioned, SARC-F is currently the most popular of the four available sarcopenia screening questionnaires. In September 2018 the Extended European Working Group on Sarcopenia in Older People (EWGSOP2) revised the criteria for sarcopenia initially published in April 2010 and recommended the application of SARC-F as a screening tool in the first step of the practical algorithm: the so-called Find-Assess-Confirm-Severity (FACS) pathway [14,15]. Furthermore, the European Union Geriatric Medicine Society, Sarcopenia Special Interest Group, has taken action to validate different language versions of this questionnaire [16].

To unambiguously determine which questionnaire is the most effective tool for sarcopenia screening, analyses are necessary to compare the diagnostic values of each of the tools against gold standards, both in community-dwelling older people and high-risk groups, i.e. hospitalised older patients and residents of nursing homes. To the best of our knowledge, the only study comparing the SARC-F, SARC-CalF, MSRA-5 and MSRA-7 questionnaires is the analysis by Yang et al. [17], performed in residents from nursing homes. No studies comparing all four tools in non-institutionalised older subjects have been published. For community-dwelling older adults, there are as few as three reports that compare the diagnostic values of the SARC-F and SARC-CalF questionnaires [9,10,18], and one analysis comparing MSRA-5, MSRA-7 and SARC-F [19]. Thus, our study aims to assess the diagnostic value of these tools and compare the obtained results to fill the research gap in this area.

We assessed the diagnostic value of four questionnaires used for the screening of sarcopenia (SARC-F, SARC-CalF, MSRA-5, MSRA-7), and compared their psychometric properties against six sets of international diagnostic criteria of sarcopenia (EWGSOP1 [14], EWGSOP2 [15], FNIH [20], AWGS [21], IWGS [22], and SCWD [23]) in community-dwelling elderly individuals from Poland.

## Materials and methods

We performed a diagnostic accuracy study from March until July 2019, for which we recruited older adults, living in the community in Poznan, one of the largest cities in Poland. The inclusion criteria were as follows: age (65 years or more), lack of cognitive impairment [defined as Abbreviated Mental Test Score (AMTS) ≥ 8 points)], the ability to take a vertical position (necessary for measuring body height and analysing body composition for the assessment of Appendicular Lean Mass), and the ability to perform a 4-m usual walking speed test. The exclusion criteria were designed based on what makes the measurement of body composition impossible (e.g., implanted artificial pacemaker or the presence of metal implants). One hundred ten persons volunteered for the study. Ten of them were excluded for the following reasons: cognitive impairment (n = 5), having a pacemaker (n = 2), physical disability preventing a 4-m usual walking speed test (n = 3).

The study protocol was approved by the Bioethics Committee of the Poznan University of Medical Sciences, Poland (approval No: 872/18). Informed consent was obtained from each subject prior to the study.

### Criterion standards for sarcopenia (gold standards)

We used six sets of international diagnostic criteria listed below as the reference standard of sarcopenia diagnosis: (1) the EWGSOP1 [14]; (2) the EWGSOP2 [15], (3) the Foundation for the National Institutes of Health (FNIH) Sarcopenia Project [20], (4) the Asia Working Group for Sarcopenia (AWGS) [21], (5) the International Working Group for Sarcopenia (IWGS) [22], and (6) the Society on Sarcopenia, Cachexia and Wasting Disorders (SCWD) [23].

According to the EWGSOP1 criteria [14], sarcopenia is defined as low muscle mass (LMM) and strength, and/or low physical performance. We used cut-off points for LMM for the Polish population defined by the ALM index and young, healthy reference population aged 18–40 years, i.e. 7.4 kg/m^2^ for men and 5.6 kg/m^2^ for women [24]. Each subject was considered to have low muscle mass if their ALM index was less than or equal to the sex-specific Polish cut-off points. The cut-off point for low handgrip strength (HGS) was <30 kg for men, <20 kg for women and the cut-off point for low physical performance was a gait speed (GS) of ≤ 0.8 m/s both sexes. According to EWGSOP2 definition [15], sarcopenia is defined as low muscle strength, ie. HGS < 27 kg for men and <16 kg for women and/or chair stand test (CST) > 15 s for both sexes and low muscle quantity (i.e. low muscle mass). To define low muscle mass, we used the same as in the EWGSOP1 algorithm sex-specific Polish cut-off points (i.e. ≤7.4 kg/m^2^ for men and ≤5.6 kg/m^2^ for women [24]). In accordance with the recommendations of FNIH [20] sarcopenia is defined as low muscle mass [appendicular lean mass (ALM)/body mass index (BMI): <0.789 for men and <0.512 for women], and weakness (HGS: <26 kg for men <16 kg for women), and slowness (GS ≤0.8 m/s for both sexes). According to the diagnostic criteria of AWGS [21], sarcopenia is defined as low muscle mass (ALM index <7.0 kg/m^2^ for men and <5.6 kg/m^2^ for women), accompanied by low muscle function (HGS < 26 kg for men and < 18 kg for women and/or GS < 0.8 m/s for both sexes). According to the IWGS criteria [22], sarcopenia is defined as an ALM index value ≤7.23 kg/m^2^ for men and ≤5.67 kg/m^2^ for women, and a GS value of <1 m/s both sexes. According to the diagnostic criteria of the SCWD [23], sarcopenia is defined as low muscle mass and low physical performance. Following the recommendations of SCWD [23], we used Polish cut-off points determined earlier from a study of healthy subjects between 20 and 30 years of age of the same ethnic group, i.e. 7.29 kg/m^2^ for men and 5.52 kg/m^2^ for women [25]. Each participant was considered to have low muscle mass if their ALM index was below or equal these sex-specific cut-off points for LMM. The cut-off point for low physical performance was a gait speed (GS) of ≤ 1.0 m/s for both sexes.

### Assessment of muscle mass

The muscle mass level was assessed in each study participant using the BIA method (InBody 120, Biospace, Seoul, South Korea). The InBody 120 is a segmental impedance device which uses a tetrapolar 8-point tactile electrode method. The device has built-in hand and foot electrodes. Ten impedance measurements are performed using two different frequencies (20 and 100 kHz) at each segment (right arm, left arm, trunk, right leg, and left leg). The subject’s identification number, age, sex and height were entered into the analyser. The analyser gives immediate and detailed results, including quantitative values of weight, BMI and other body composition parameters. Only segmental lean mass data were used for further analysis for calculating the Appendicular Lean Mass (ALM) index. The ALM index [the ratio of ALM (kg) and squared height (m^2^)] was calculated for each subject. Height assessment was performed by means of a mobile stadiometer (Tanita, Poznan, Poland).

### Assessment of muscle strength

Muscle strength was assessed by handgrip strength with a dynamometer (Saehan, Changwon, South Korea). Participants performed the handgrip strength test in a sitting position, with arms bent to 90 degrees in the elbow and shoulder joint. Both the left and right arms were measured twice. The results were recorded in kilograms (kg). The mean value of all measurements was used as the final score for each individual. We also assessed lower limb strength using The Chair Stand Test (CST), which was necessary to apply the EWGSOP2 criteria [15]. Each subject was asked to rise five times from a chair with arms folded across the chest, and the time needed to complete the test was measured. The results were recorded in seconds (s).

### Assessment of physical performance

Physical performance was assessed using the 4-m usual walking speed test. This test measures the walking pace at the distance of 4 meters–subjects are asked to walk the course at their usual gait speed. Time taken to perform the walk was recorded, and the result expressed as meters per second. If necessary, canes or walkers were permitted during this test.

### Screening for sarcopenia

The risk of sarcopenia was evaluated in each studied subject using four questionnaires: SARC-F, SARC-CalF, MSRA-7, and MSRA-5.

#### The SARC-F questionnaire

The SARC-F [4] examines five domains: 1) strength, 2) assistance with walking, 3) rising from a chair, 4) climbing stairs, and 5) falls, scored from 0 to 2. A score of ≥4 out of the maximum of 10 points indicates a risk of sarcopenia.

#### The SARC-CalF questionnaire

SARC-CalF [9] is composed of six items, the first five items being and scored the same as the SARC-F and the sixth additional item being the calf circumference item (CC; measurement of the right calf in standing position).The measurement of CC requires the use of an anthropometric measuring tape. The CC score is interpreted separately for each gender. The cut-off points of CC are 34 and 33 cm for men and women, respectively. The CC item is scored as 0 points if its value is above the cut-off points and as 10 if its value is below or equals the cut-off points. A score of ≥11 points indicates a risk of sarcopenia.

#### The MSRA questionnaires

The full version of the MSRA questionnaire (MSRA-7) examines seven domains including 1) age, 2) hospitalisation in the last year, 3) level of activity, 4) regularity of meals, 5) daily dairy consumption, 6) protein intake, and 7) weight loss >2 kg in the last year. The short version (MSRA-5) excludes dairy and protein consumption. A total score of MSRA-7 ≤30 and MSRA-5 ≤45 points indicates a risk of sarcopenia.

### Covariates

#### Assessment of cognitive function

Cognitive functions were assessed with the Abbreviated Mental Test Score (AMTS) [26]. The test is composed of 10 questions. Every subject scores 1 point for a correct answer and 0 points for an incorrect answer or no answer. Individuals who score 8 points or more are considered cognitively intact. Only subjects who scored at least 8 points were qualified for this study.

#### Nutritional assessment

To evaluate the nutritional condition of the participants, the Mini Nutritional Assessment–Short Form (MNA-SF) was used [27]. The MNA-SF is composed of 6 items and assesses decrease in food intake, weight loss, mobility, psychological stress or acute disease, neuropsychological problems (dementia or depression), and BMI. The maximal score of the MNA-SF is 14 points. A score below 7 points indicates malnutrition, 8–11 points–a risk of malnutrition, and 12 points or more–normal nutritional status.

#### Assessment of independence in activities of daily living

Independence in basic and instrumental activities of daily living was assessed with the Katz scale and Lawton scale, respectively [28,29]. The Katz scale is composed of six tasks: bathing, dressing and undressing, toileting, transferring from and to bed, and continence (bowel and bladder), scored as 0, 0,5 or 1. According to the ADL score, participants were classified as: dependent (0–2 points), partially dependent (3–4 points) and independent (5–6 points).

The Lawton scale assesses performance in eight dimensions: the ability to use the telephone, ability to use different modes of transportation, shopping, food preparation, housekeeping (doing laundry and cleaning), control over one’s own medications and ability to handle finances, scored from 1 to 3. The maximum score is 24 points. As far as the Lawton scale is concerned, there are no cut-off points that would define different levels of independence. However, it does allow for profiling the patient’s needs for assistance or care, as lower results indicate a higher level of dependence.

### Statistical analysis

Statistical analysis was performed using the STATISTICA 12.0 package (StatSoft, Poland). Continuous data were presented as mean ± SD and compared using a Student’s t-test or the Cochran-Cox test or Mann–Whitney test as appropriate. Categorical variables were expressed as number (percentage) and compared with the χ2 test (applying the Yates correction when necessary). The EWGSOP1 [14], EWGSOP2 [15], FNIH [20], AWGS [21], IWGS [22], and SCWD criteria [23] were used as the criterion standards for sarcopenia (gold standards). Next, sensitivity, specificity, positive predictive value (PPV) and negative predictive value (NPV) of SARC-F, SARC-CalF, MSRA-5 and MSRA-7 were calculated. The sensitivity is the proportion of subjects actually presenting sarcopenia (based on the gold standard), having been correctly identified as sarcopenic using the screening test (i.e., positive screening test). The specificity represents the proportion of individuals who do not have sarcopenia (based on the gold standard), which were correctly identified as non-sarcopenic using the screening test (i.e., negative screening test). The PPV is a measure of the probability of presenting sarcopenia in case of a positive screening test; in turn, the NPV represents the probability of not having sarcopenia in case of a negative screening test [30]. All of these parameters were specified with 95% confidence intervals (CI). The ROC curve was used for comparing the overall diagnostic accuracy. Areas under the ROC curve (AUC) with 95%CI were calculated. A higher AUC corresponded to a higher overall diagnostic accuracy. It was assumed that the AUC values >0.9, 0.7 to 0.9, and 0.5 to 0.7 corresponded to the high, moderate and low diagnostic accuracy of the screening test, respectively [10,31]. The areas under the ROC curve were compared using the Hanley-McNeil non-parametric method [32,33].

## Results

### Characteristics of the study group

The analysis included a total of 100 community-dwelling volunteers aged 65 years and older (age range: 65–93 years); 21% of them were male. Table 1 shows the characteristics of the whole study group by gender. The mean age of women and men was comparable (p>0.05). Comparing the women to men, women were statistically significantly shorter (156.9±6.0 vs 173.5±6.6 cm, p<0.001) and thinner (65.9±13.8 vs 78.1±11.7 kg, p<0.001) but had similar BMI to men (26.8± 5.6 vs 25.9±3.6 kg/m^2^, p>0.05).

**Table 1:** Characteristics of the whole study population and according to gender.

Characteristics	Total (n = 100)	Men (n = 21)	Women (n = 79)	p
Age (years)^a^	74.5 (6.9)	74.8 (7.2)	74.4 (6.8)	0.8092
Age cohort^b^				
65–74 yrs	55 (55.0)	12 (57.1)	43 (54.4)	0.8243
75 yrs or more	45 (45.0)	9 (42.9)	36 (45.6)	
Height (cm) ^a^	160.4 (9.1)	173.5 (6.6)	156.9 (6.0)	0.0000
Weight (kg) ^a^	68.5 (4.2)	78.1 (11.7)	65.9 (13.8)	0.0003
BMI (kg/m^2^) ^a^	26.6 (5.3)	25.9 (3.6)	26.8 (5.6)	0.4010
Low BMI^b^				
Yes	17 (17.0)	2 (9.5)	15 (19.0)	0.4843
No	83 (83.0)	19 (90.5)	64 (81.0)	
MNA-SF score ^a^	12.3 (3.6)	12.9 (4.3)	12.2 (3.4)	0.6638
MNA-SF, status^b^				
Malnutrition	7 (7.0)	1 (4.8)	6 (7.6)	0.4429
Risk of malnutrition	23 (23.0)	3 (14.3)	20 (25.3)	
Normal nutritional status	70 (70.0)	17 (80.9)	53 (67.1)	
ADL score ^a^	5.7 (0.4)	5.9 (0.3)	5.7 (0.5)	0.0443
ADL, status^b^				
Independent	98 (98.0)	21 (100.0)	77 (97.5)	0.8884
Partially dependent	2 (2.0)	0 (0.0)	2 (2.5)	
Dependent	0 (0.0)	0 (0.0)	0 (0.0)	
IADL score ^a^	25.6 (2.7)	26.0 (1.7)	25.5 (2.9)	0.6749
AMTS score ^a^	9.2 (0.6)	9.2 (0.7)	9.2 (0.6)	0.9849
Number of regular drugs^b^				
0–3	43 (43.0)	9 (42.9)	34 (43.0)	0.9881
4 or more	57 (57.0)	12 (57.1)	45 (57.0)	
Handgrip strength ^a^	21.9 (8.0)	32.8±8.0	19.0 (5.0)	0.0000
Gait speed ^a^	1.0 (0.3)	1.0 (0.3)	0.9 (0.3)	0.2231
Chair stand test (s)^a^**	12.73 (4.4)	12.6 (4.3)	12.77 (4.5)	0.7959
ALM (kg) ^a^	17.3 (4.2)	23.4 (2.6)	15.7 (2.8)	0.0000
ALM index (kg/m^2^) ^a^	6.6 (1.0)	7.7 (0.6)	6.4 (0.9)	0.0000
Calf circumference ^a^	35.4 (3.7)	35.9 (2.3)	35.3 (4.0)	0.4014
SARC-F score ^a^	1.8 (1.9)	1.2 (1.1)	2.0 (2.1)	0.2172
SARC-CalF score ^a^	4.4 (5.1)	3.5 (4.9)	4.6 (5.1)	0.2911
MSRA-5 score ^a^	44.7 (11.8)	44.0 (12.8)	44.9 (11.6)	0.8372
MSRA-7 score ^a^	28.2 (7.1)	27.1 (7.3)	28.4 (7.1)	0.4710

^a^ Data are presented as mean (standard deviation)

^b^ Data are presented as n (%)

* low BMI, i.e. <20 if < 70 years, or <22 if ≥70 years

** n = 94, excluded six women who were unable to complete the chair stand test due to low back pain

Abbreviations: BMI, Body Mass Index; MNA-SF, Mini Nutritional Assessment—Short Form; ADL, Activity of Daily Living; IADL, Instrumental Activity of Daily Living; AMTS, Abbreviated Mental Test Score; ALM, appendicular lean mass; MSRA, Mini Sarcopenia Risk Assessment

Almost 1/5 of the study group had low BMI, and this feature was observed twice as often in women. Almost 1/3 of the participants had poor nutritional status (i.e. malnutrition or risk of malnutrition). Almost all participants were independent according to the ADL scale. The group of studied women had a statistically significantly lower score for the activities of daily living (5.7±0.5 vs 5.9±0.3 points, p<0.05, respectively), but not for the instrumental activities of daily living (25.5±2.9 vs 26.0±1.7 points, p>0.05). More than half of the participants took four or more drugs a day–this affected women and men equally.

An assessment of muscle function showed that men were stronger than women (32.8±8.0 vs 19.0±5.0 kg, p<0.001, respectively). However, both groups were characterised by a similar level of physical performance assessed by the 4-m usual walking speed test. Lower appendicular lean mass (ALM) was found in the studied women compared to men (15.7±2.8 vs 23.4±2.6 kg, respectively, p<0.001). The ALM index was statistically significantly lower in women than in men as well. Table 1 also contains the mean values for the studied sarcopenia screening questionnaires for the all study group and according to gender. No statistically significant differences were noted.

Table 2 summarises the answers given to the questions from the SARC-F questionnaire, with additional calf circumference measurement (for the SARC-CalF questionnaire). Almost half of the respondents reported difficulties with lifting and carrying a weight of 5 kg, and this problem was statistically significantly more frequently reported by women (p<0.05). 1/3 of the participants indicated problems standing up from a chair or bed. Almost a quarter of the study group reported problems climbing a flight of 10 stairs and experienced at least one fall in the past year. About 15% of participants declared moderate or major difficulties in walking across a room. Calf circumference below the recommended cut-off points (≤ 33 cm for women and ≤ 34 cm for men) was observed in more than 1/4 of the subjects (comparably often in the group of women and men).

**Table 2:** The characteristics of answers given to the questions from the SARC-F combined with calf circumference of the whole study population and according to gender.

SARC-F components	Total (n = 100)	Men (n = 21)	Women (n = 79)	p
Q1. Strength—difficulty lifting and carrying about 5 kg				
None	51 (51.0)	16 (76.2)	35 (44.3)	0.0292
Some	28 (28.0)	3 (14.3)	25 (31.6)	
A lot or unable	21 (21.0)	2 (9.5)	19 (24.1)	
Q2. Assistance in walking—difficulty walking across a room				
None	86 (86.0)	19 (90.5)	67 (84.8)	0.4653
Some	11 (11.0)	2 (9.5)	9 (11.4)	
A lot, use aids, or unable	3 (3.0)	0 (0.0)	3 (3.8)	
Q3. Rise from a chair—difficulty transferring from a chair or bed				
None	69 (69.0)	16 (76.2)	53 (67.1)	0.2723
Some	26 (26.0)	5 (23.8)	21 (26.6)	
A lot or unable without help	5 (5.0)	0 (0.0)	5 (6.3)	
Q4. Climb stairs—difficulty climbing a flight of 10 stairs				
None	77 (77.0)	16 (76.2)	61 (77.2)	0.2436
Some	18 (18.0)	5 (23.8)	13 (16.5)	
A lot or unable	5 (5.0)	0 (0.0)	5 (6.3)	
Q5. Falls—times fallen in the past year				
None	77 (77.0)	16 (76.2)	61 (77.2)	0.3339
1–3 falls	19 (19.0)	5 (23.8)	14 (17.7)	
≥ 4 falls	4 (4.0)	0 (0.0)	4 (5.1)	
Calf circumference				
K > 33 cm / M >34 cm	74 (74.0)	16 (76.2)	58 (73.4)	0.9821
K ≤ 33 cm / M ≤ 34 cm	26 (26.0)	5 (23.8)	21 (26.6)	

Data are presented as n (%); Q- question

Table 3 presents the answers given to the questions from the MSRA questionnaire. Over 2/3 of the study group was aged 70 years or above. More than 1/3 of participants reported that they had been treated in hospital at least once in the last year. A similar percentage indicated that they lost weight >2kg in the last year, and this issue affected women 2.5 times more often than men (p = 0.0621 was close to statistical significance). About 1/5 of respondents skip a meal up to twice per week, and a quarter of the participants in this analysis did not consume protein-rich products (e.g. meat, eggs, legumes, milk or dairy products). 1/5 of the study group was unable to walk more than 1000 metres.

**Table 3:** The characteristics of answers given to the questions from the Mini Sarcopenia Risk Assessment (MSRA) questionnaire (full version) of the whole study population and according to gender.

MSRA components	Total (n = 100)	Men (n = 21)	Women (n = 79)	p
Q1.Age				
≥ 70 yrs	71 (71.0)	15 (71.4)	56 (70.9)	0.9611
< 70 yrs	29 (29.0)	6 (28.6)	23 (29.1)	
Q2. Number of hospital treatment in the last year				
Yes, more than once	14 (14.0)	3 (14.3)	11 (13.9)	0.3699
Yes, once	22 (22.0)	7 (33.3)	15 (19.0)	
No	64 (64.0)	11 (52.4)	53 (67.1)	
Q3. Level of physical activity				
Able to walk less than 1000 m	19 (19.0)	5 (23.8)	14 (17.7)	0.7496
Able to walk more than 1000 m	81 (81.0)	16 (76.2)	65 (82.3)	
Q4. Regular consumption three meals a day				
No, up to twice a week I skip a meal	18 (18.0)	5 (23.8)	13 (16.5)	0.4478
Yes	82 (82.0)	16 (76.2)	66 (83.5)	
Q5. Consumption of dairy products				
Yes, but not every day	26 (26.0)	8 (38.1)	18 (22.8)	0.1671
Yes, at least once a day	74 (74.0)	13 (61.9)	61 (77.2)	
Q6. Consumption of proteins				
Yes, but not every day	24 (24.0)	5 (23.8)	19 (24.1)	0.9816
Yes, at least once a day	76 (76.0)	16 (76.2)	60 (75.9)	
Q7. Weight loss in the last year				
> 2 kg	30 (30.0)	3 (14.3)	27 (34.2)	0.0621
no or ≤ 2 kg	70 (70.0)	18 (85.7)	52 (65.8)	

Data are presented as n (%); Q- question

### Prevalence of sarcopenia

The frequency of sarcopenia varied from 17% to 72%, depending on the questionnaire used (Table 4). SARC-F identified the lowest number of subjects with a risk of sarcopenia (17 persons, including 16 women), whereas MSRA-7 –the highest (72 persons, including 56 women). A large spread of results was observed when we used six sets of international diagnostic criteria for sarcopenia (Table 4). The lowest percentage of patients with sarcopenia (10%) was diagnosed with FNIH criteria. In contrast, the highest percentage of patients with sarcopenia (20%) was identified by the EWGSOP1 criteria. The same frequency of sarcopenia was recognised by IWGS and SCWD criteria (n = 12). Regardless of the type of screening test or diagnostic criteria for sarcopenia, the condition was found to be more prevalent in women than in men. However, due to a low number of men with sarcopenia in our study, the statistical analysis including gender was not performed.

**Table 4:** Prevalence of sarcopenia according to four different questionnaires and six sets of international diagnostic criteria of sarcopenia.

Characteristics		Total (n = 100)	Men (n = 21)	Women (n = 79)	p
SARC-F classification	Sarcopenia	17 (17.0)	1 (4.8)	16 (20.3)	0.1761
	Non-sarcopenia	83 (83.0)	20 (95.2)	63 (79.7)	
SARC-CalF classification	Sarcopenia	20 (20.0)	4 (19.0)	16 (20.3)	0.8539
	Non-sarcopenia	80 (80.0)	17 (81.0)	63 (79.7)	
MSRA-5 classification	Sarcopenia	56 (56.0)	11 (52.4)	45 (57.0)	0.8977
	Non-sarcopenia	44 (44.0)	10 (47.6)	34 (43.0)	
MSRA-7 classification	Sarcopenia	72 (72.0)	16 (76.2)	56 (70.9)	0.8354
	Non-sarcopenia	28 (28.0)	5 (23.8)	23 (29.1)	
FNIH classification	Sarcopenia	10 (10.0)	1 (4.8)	9 (11.4)	0.6234
	Non-sarcopenia	90 (90.0)	20 (95.2)	70 (88.6)	
AWGS classification	Sarcopenia	16 (16.0)	1 (4.8)	15 (19.0)	0.2129
	Non-sarcopenia	84 (84.0)	20 (95.2)	64 (81.0)	
EWGSOP1 classification	Sarcopenia	20 (20.0)	5 (23.8)	15 (19.0)	0.8539
	Non-sarcopenia	80 (80.0)	16 (76.2)	64 (81.0)	
EWGSOP2 classification	Sarcopenia*	17 (17.0)	5 (23.8)	12 (15.2)	0.5433
	Non-sarcopenia	83 (83.0)	16 (76.2)	67 (84.8)	
IWGS classification	Sarcopenia	12 (12.0)	1 (4.8)	11 (13.9)	0.4409
	Non-sarcopenia	88 (88.0)	20 (95.2)	68 (86.1)	
SCWD classification	Sarcopenia	12 (12.0)	0 (0.0)	12 (15.2)	0.1270
	Non-sarcopenia	88 (88.0)	21 (100.0)	67 (84.8)	

Data are presented as n (%)

* sarcopenia confirmed

Abbreviations: MSRA, Mini Sarcopenia Risk Assessment; EWGSOP1, the European Working Group on Sarcopenia in Older People 1; EWGSOP2, extended group for the European Working Group on Sarcopenia in Older People 2; FNIH, the Foundation for the National Institutes of Health; AWGS, Asian Working Group on Sarcopenia; IWGS, the International Working Group on Sarcopenia; SCWD, the Society on Sarcopenia, Cachexia and Wasting Disorders

### Diagnostic value of the analysed questionnaires for sarcopenia screening

Concerning the six criterion standard for sarcopenia (gold standards) used in the study, the sensitivity of the compared tools varied in the following ranges: SARC-F 35.0–90.0%, SARC-CalF 20.0–75.0%, MSRA-5 64.7–90.0%, and MSRA-7 76.5–91.7% (Table 5). The specificity ranges were as follows: SARC-F 86.9–91.1%, SARC-CalF 80.0–90.0%, MSRA-5 45.8–48.8%, and MSRA-7 28.9–31.0%. The range of results for PPV varied from 10% (for SARC-CalF against FNIH) to 60% (for SARC-CalF against EWGSOP1), whereas for NPV–from 84.3% (for SARC-F against EWGSOP1) to 98.8% (for SARC-F against to FNIH). The AUCs of SARC-F, SARC-CalF, MSRA-5, and MSRA-7 ranged from 0.655–0.882, 0.711–0.874, 0.618–0.782 and 0.588–0.711 respectively. SARC-F had the largest AUC of the four analysed tools but only against FNIH criteria (0.882), and this result indicates nearly an excellent level of diagnostic accuracy. In turn, SARC-CalF had AUC >0.7 but <0.9 against all six gold standards, which suggests a moderate level of diagnostic accuracy. MSRA-7 had the smallest AUC of all the questionnaires against to the EWGSOP2 criteria (0.588). This tool had a similarly small AUC with respect to the EWGSOP1 (0.608), FNIH (0.619) and AWGS (0.668) criteria. These results indicate low diagnostic accuracy of this tool. For the remaining two criteria, the AUC of MSRA-7 marginally exceeded the value of 0.7. A slightly larger AUC value was found for MSRA-5, with results obtained for this parameter ranging from 0.618 (for EWGSOP2) to 0.782 (for SCWD). MSRA-5 proved to be a slightly more effective tool than MSRA-7, as it exceeded the value of 0.7 for three reference criteria.

**Table 5:** Sensitivity, Specificity, Positive and Negative Predictive Values and Receiver Operating Curve Model of the SARC-F, SARC-CalF, MSRA-5 and MSRA-7 questionnaires against six sets of international diagnostic criteria of sarcopenia in the whole study population.

	Sensitivity (%)	Specificity (%)	PPV (%)	NPV (%)	AUC
**FNIH**					
SARC-F	90.0 (55.5–99.8)	91.1 (83.2–96.1)	52.9 (36.0–69.2)	98.8 (92.7–99.8)	0.882 (0.726–1.000)^b^^,^^c^^,^^d^
SARC-CalF	20.0 (2.5–55.6)	80.0 (70.3–87.7)	10.0 (2.9–29.1)	90.0 (86.7–92.6)	0.711 (0.556–0.865)^a^
MSRA-5	90.0 (55.5–99.8)	47.8 (37.1–58.6)	16.1 (12.6–20.3)	97.7 (86.8–99.6)	0.669 (0.539–0.799)^a^
MSRA-7	90.0 (55.5–99.8)	30.0 (20.8–40.6)	12.5 (10.0–15.5)	96.4 (80.4–99.4)	0.619 (0.463–0.775)^a^
**AWGS**					
SARC-F	37.5 (15.2–64.6)	86.9 (77.8–93.3)	35.3 (14.2–61.7)	88.0 (79.0–94.1)	0.655 (0.496–0.814)^b^
SARC-CalF	62.5 (35.4–84.8)	88.1 (79.2–94.1)	50.0 (27.2–72.8)	92.5 (84.4–97.2)	0.786 (0.636–0.936)^a^
MSRA-5	81.3 (54.4–96.0)	48.8 (37.7–60.0)	23.2 (13.0–36.4)	93.2 (81.3–98.6)	0.728 (0.582–0.873)
MSRA-7	87.5 (61.7–98.4)	31.0 (21.3–42.0)	19.4 (11.1–30.5)	92.9 (76.5–99.1)	0.668 (0.519–0.816)
**EWGSOP1**					
SARC-F	35.0 (15.4–59.2)	87.5 (78.2–93.8)	41.2 (18.4–67.1)	84.3 (79.0–94.1)	0.658 (0.513–0.802)^b^
SARC-CalF	60.0 (36.1–80.9)	90.0 (81.2–95.6)	60.0 (36.1–80.9)	90.0 (81.2–95.6)	0.816 (0.691–0.940)^a^^,^^d^
MSRA-5	70.0 (45.7–88.1)	47.5 (36.2–59.0)	25.0 (14.4–38.4)	86.4 (72.6–94.8)	0.653 (0.510–0.796)
MSRA-7	80.0 (56.3–94.3)	30.0 (20.3–41.3)	22.2 (13.3–33.6)	85.7 (67.3–96.0)	0.608 (0.467–0.750)^b^
**EWGSOP2**					
SARC-F	41.2 (18.4–67.1)	88.0 (79.0–94.1)	41.2 (18.4–67.1)	88.0 (79.0–94.1)	0.719 (0.579–0.859)
SARC-CalF	64.7 (38.3–85.8)	89.2 (80.4–94.9)	55.0 (31.5–76.9)	92.5 (84.4–97.2)	0.792 (0.648–0.936)^d^
MSRA-5	64.7 (38.3–85.8)	45.8 (34.8–57.1)	19.6 (10.2–32.4)	86.4 (72.6–94.8)	0.618 (0.460–0.776)
MSRA-7	76.5 (50.1–93.2)	28.9 (19.5–39.9)	18.1 (10.0–28.9)	85.7 (67.3–96.0)	0.588 (0.431–0.746)^b^
**IWGS**					
SARC-F	50.0 (21.1–78.9)	87.5 (78.7–93.6)	35.3 (14.2–61.7)	92.8 (84.9–97.3)	0.732 (0.555–0.908)
SARC-CalF	66.7 (34.9–90.1)	86.4 (77.4–92.8)	40.0 (19.1–63.9)	95.0 (87.7–98.6)	0.846 (0.704–0.988)
MSRA-5	83.3 (51.6–97.9)	47.7 (37.0–58.6)	17.9 (8.9–30.4)	95.5 (84.5–99.4)	0.767 (0.614–0.920)
MSRA-7	91.7 (61.5–99.8)	30.7 (21.3–41.4)	15.3 (7.9–25.7)	96.4 (81.7–99.9)	0.711 (0.556–0.866)
**SCWD**					
SARC-F	50.0 (21.1–78.9)	87.5 (78.7–93.6)	35.3 (14.2–61.7)	92.8 (84.9–97.3)	0.719 (0.543–0.895)^b^
SARC-Cal-F	75.0 (42.8–94.5)	87.5 (78.7–93.6)	45.0 (23.1–68.5)	96.3 (89.4–99.2)	0.874 (0.735–1.000)^a^
MSRA-5	83.3 (51.6–97.9)	47.7 (37.0–58.6)	17.9 (8.9–30.4)	95.5 (84.5–99.4)	0.782 (0.642–0.921)
MSRA-7	91.7 (61.5–99.8)	30.7 (21.3–41.4)	15.3 (7.9–25.7)	96.4 (81.7–99.9)	0.711 (0.556–0.866)

Data are presented with the 95% CI in parenthesis

a–Significantly different with SARC-F (p<0.05)

b–Significantly different with SARC-Cal-F (p<0.05)

c–Significantly different with MSRA-5 (p<0.05)

d–Significantly different with MSRA-7 (p<0.05); Abbreviations: PPV, Positive Predictive Values; NPV, Negative Predictive Values; AUC area under the curve; MSRA, Mini Sarcopenia Risk Assessment; EWGSOP1, the European Working Group on Sarcopenia in Older People; EWGSOP2, extended group for the European Working Group on Sarcopenia in Older People; FNIH, the Foundation for the National Institutes of Health; AWGS, Asian Working Group on Sarcopenia; IWGS, the International Working Group on Sarcopenia; SCWD, the Society on Sarcopenia, Cachexia and Wasting Disorders

**Fig 1:**
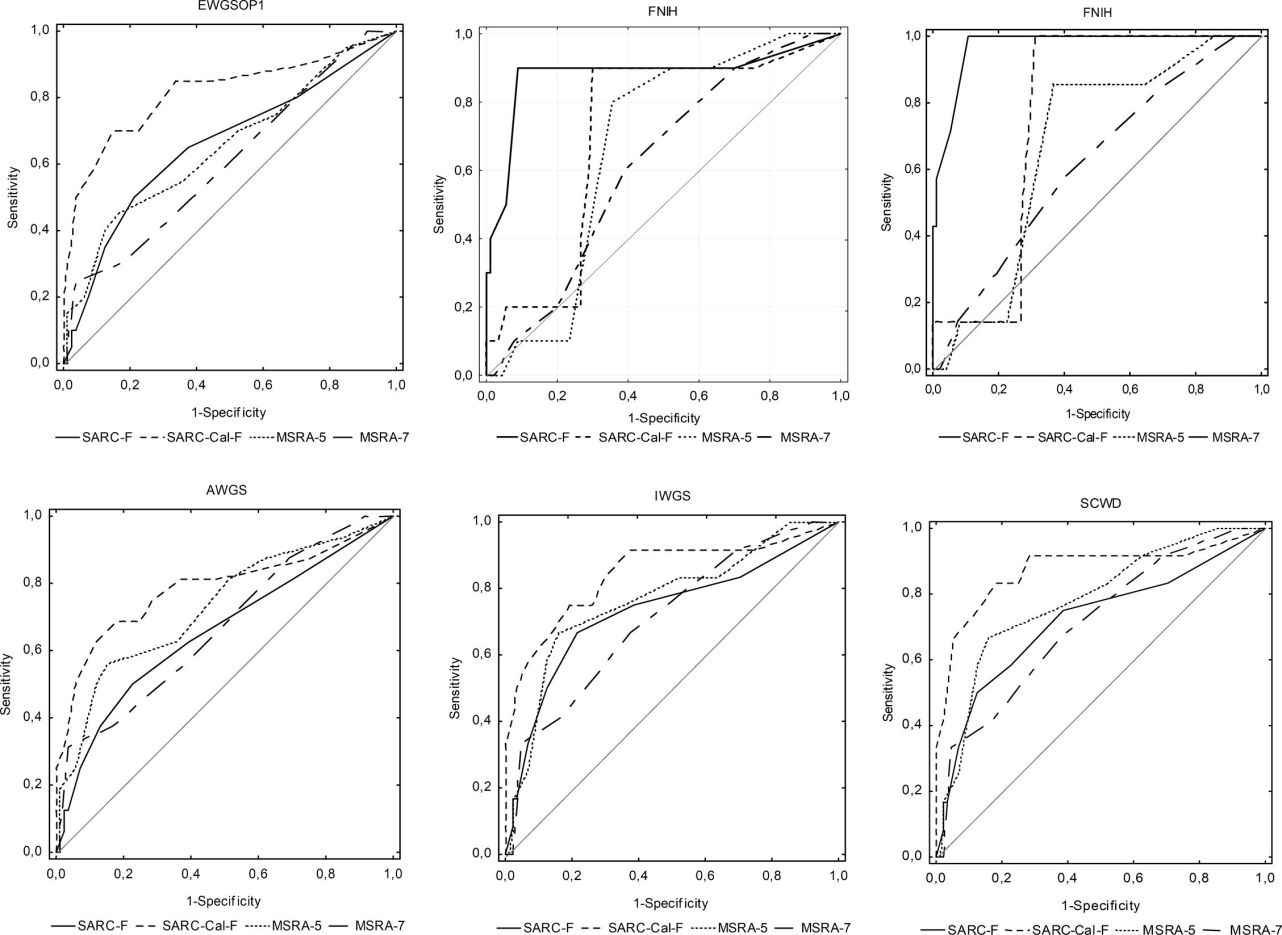
The ROC curves of SARC-F, SARC-CalF, MSRA-7 and MSRA-5 against six sets of international diagnostic criteria of sarcopenia.

ROC curves of the four screening questionnaires against six sets of international diagnostic criteria of sarcopenia are shown in Fig 1.

## Discussion

Since sarcopenia has serious health implications, early detection of the condition through screening in the general population is an important task. Several sarcopenia screening tools are currently available, but there have scarcely been any studies to determine which of them has superior efficacy in detecting sarcopenia in community-dwelling older people. Our analysis fills this gap. To the best of our knowledge, the results reported in this paper are the first analysis of this type in Caucasian community-dwelling older adults (from Central and Eastern Europe).

The purpose of screening is to detect sarcopenia in as early a stage as possible, so that early therapeutic intervention is possible. However, the screening results must be verified with a subsequent professional diagnosis, due to the risk of a false positive. Ideal screening tools should thus have reasonably high sensitivity and specificity, and an AUC value above 0.7 [10,34]. The larger the AUC, the better the overall diagnostic accuracy [10,18]. In our analysis, SARC-F was shown to have the highest sensitivity (90.0%), high specificity (91.1%) and large AUC (0.882), but only against the FNIH criteria [20]. At the same time, the FNIH criteria recognised the lowest percentage of people with sarcopenia (ten persons). In turn, based on the literature, SARC-F had low sensitivity, but high specificity and overall good diagnostic accuracy [5–8]. That was confirmed by our study for five out of six sets of international diagnostic criteria of sarcopenia (except the results related to the FNIH criteria).

In response to the reported unsatisfactory sensitivity of SARC-F, Barbosa-Silva et al. proposed an extension of the questionnaire for sarcopenia screening, called SARC-CalF [9]. In an analysis of 179 older Brazilians, a comparison of SARC-CalF against SARC-F showed the former to have higher sensitivity (66.7% vs 33.3%, respectively) and AUC (0.736 vs 0.592, respectively), and comparable specificity (82.9% vs 84.2%, respectively). Only the EWGSOP1 criteria were used as the gold reference standard in this analysis. SARC-CalF differs from SARC-F by the evaluation of an additional parameter (calf circumference). This measurement should be regarded as a surrogate measure for muscle mass, which, in addition to low muscle strength, represents an essential component of sarcopenia. In our analysis, SARC-CalF, depending on the reference standard, exhibited highly varied sensitivity (20.0 to 75.0%), a less varied specificity (80.0 to 90.0%) and moderate diagnostic accuracy (AUC: from 0.711 to 0.874). SARC-CalF was shown to have the lowest sensitivity against the FNIH criteria (only 20.0%), with sarcopenia identified in only two older person, even though in relation to the same criteria SARC-F exhibited 90.0% sensitivity and detected this condition in 9 out of 10 subjects. Such discrepancies may be attributed to obesity (BMI>30 kg/m^2^) and large calf circumference in six of these ten individuals, which exceeded the CC cut-off points in the SARC-CalF questionnaire. It should be noted here that, according to the SARC-CalF questionnaire, a score of ≥11 points already indicates a risk of sarcopenia. As a consequence, if the calf circumference is small (≤ 33 for women and ≤ 34 cm for men, which gives 10 points), a slight deterioration in one of the other five evaluated domains is sufficient to be screened as sarcopenic. Accordingly, if large deficits are present in those five domains, the maximum score of 10 points can be obtained, but that alone is not enough to detect sarcopenia with SARC-CalF. In addition, Mohd Nawi et al. stressed that calf circumference measurements might be unreliable in many older adults, due to peripheral oedemas and peripheral vascular disease [2]. In our analysis, obesity was a confounding factor. Obesity does not exclude the coexistence of sarcopenia (i.e. sarcopenic obesity) but often masks low muscle mass [35]. Also, Yang et al. reported that using SARC-CalF may bear a risk of masking sarcopenia in older subjects with obesity [17].

The literature lists just one study in which the diagnostic values of the MSRA-5 and MSRA-7 questionnaires were compared with SARC-F in community-dwelling elderly individuals [19]. In this analysis, conducted by Yang et al., 384 elderly Chinese individuals were included, in which only one gold standard was used–the AWGS criteria. In contrast to our results, they showed a similar frequency of sarcopenia risk when using both the MSRA-7 and MSRA-5 questionnaires (34.4% and 39.0% respectively). In turn, SARC-F identified sarcopenia risk in 12.2%. Unfortunately, the possible causes of these discrepancies were not discussed by the authors. Similarly to our results, MSRA-5 showed higher sensitivity, specificity and AUC than MSRA-7, and SARC-F had much lower sensitivity but higher specificity than both MSRA-5 and MSRA-7. However, in the study by Yang et al. [19], MSRA-5 and SARC-F had similar overall diagnostic accuracy, which is not consistent with our results. It is worth pointing out that MSRA is based on low muscle mass risk factors, whereas SARC-F is based on the symptoms of sarcopenia, focusing on parameters related to the assessment of muscle strength. It should also be noted that four out of seven questions from the MSRA-7 questionnaire address issues related to the problem of malnutrition in old age (skipping meals, inadequate protein intake and dairy products consumption, weight loss). The use of MSRA-7 in our study group indicated a risk of sarcopenia in almost 3/4 respondents, while MSRA-5 (version without two questions about protein intake and dairy products)–in over 1/2 of them. In both cases, the indicated percentage of respondents with possible sarcopenia seems overestimated, especially so since the prevalence of sarcopenia in Poland is below 13% [36]. These results may be affected by the nutritional status of the respondents (almost 1/3 of them had poor nutritional status). In Poland, almost every second older person presents inadequate nutritional status, as demonstrated by the Polsenior study (representative of the Polish population) [37]. In addition, many of our respondents’ answers indicated a poorly balanced diet (i.e. irregular consumption of protein-rich products and/or skipping main meals). If the intake of calories and protein is low, it may contribute to weight loss and protein-energy malnutrition. In turn, malnutrition increases the risk of sarcopenia, as noted in 2012 by Vandewounde et al. who introduced the concept of Malnutrition-Sarcopenia Syndrome [38]. Moreover, in the original version of the MSRA-7 and MSRA-5 questionnaires, to have a positive screening result, it is enough to be aged 70 or over and lose weight >2 kg in the last year, or be hospitalised in the previous year. Many of our subjects met these conditions, but after using various diagnostic algorithms for sarcopenia, it turned out that they did not have it. We think that the cut-off points for MSRA-7 and MSRA-5 proposed by Rossi et al. [12] (≤ 30 points and ≤ 45 points, respectively) may not be suitable for populations similar to the Polish one.

Our study has some limitations. Firstly, a relatively small group of men (n = 21) was included in this analysis–this is mainly due to the feminisation of old age in Poland and the fact that older men are less likely to volunteer for research. Moreover, due to a low number of men with sarcopenia in our study, the comparative analysis for sarcopenia prevalence according to gender was not performed. Secondly, in our study, we collected neither the socio-demographic data (i.e., marital status, living alone, level of education) nor information on the number of chronic diseases or those potentially related to sarcopenia. Thirdly, we used the BIA method for the assessment of ALM instead of CT, MRI or DEXA, which are considered more precise but are hardly available in Poland. Moreover, BIA is free of x-ray exposure and seems to be a more practical (because analysers are portable) and inexpensive choice. Moreover, some international groups, such as EWGSOP1 [14], EWGSOP2 [15], AWGS [21], recommended BIA as an alternative option for muscle measurement.

A strong point of our analysis is that we were the first to use all currently available sets of international diagnostic criteria for sarcopenia as a gold standard [there are six of them, developed independently by European Working Group on Sarcopenia in Older People 1 (EWGSOP1) [14], European Working Group on Sarcopenia in Older People 2 (EWGSOP2) [15], Foundation for the National Institutes of Health (FNIH) Sarcopenia Project [20], Asia Working Group for Sarcopenia (AWGS) [21], the International Working Group for Sarcopenia (IWGS) [22], and Society on Sarcopenia, Cachexia and Wasting Disorders (SCWD)[23].

## Conclusions

Based on our analysis, the standard sarcopenia screening questionnaires deliver contradictory results in many practically occurring cases. It appears that SARC-CalF is an optimal choice for screening sarcopenia in community-dwelling older adults. However, the SARC-CalF may be inappropriate for use in obese subjects (those who often present a large calf circumference). The original cut-off points for the MSRA questionnaires may not be suitable for countries that have a high proportion of older people with poor nutritional status and inadequate diet. Perhaps, for such populations, it would be justified to set new cut-off points.
